# Dietary Diversity, Diet Cost, and Incidence of Type 2 Diabetes in the United Kingdom: A Prospective Cohort Study

**DOI:** 10.1371/journal.pmed.1002085

**Published:** 2016-07-19

**Authors:** Annalijn I. Conklin, Pablo Monsivais, Kay-Tee Khaw, Nicholas J. Wareham, Nita G. Forouhi

**Affiliations:** 1 Medical Research Council Epidemiology Unit, University of Cambridge School of Clinical Medicine, Institute of Metabolic Science, Cambridge Biomedical Campus, Cambridge, United Kingdom; 2 WORLD Policy Analysis Center, UCLA Fielding School of Public Health, Los Angeles, California, United States of America; 3 Department of Public Health and Primary Care, University of Cambridge, Cambridge, United Kingdom; Stanford University, UNITED STATES

## Abstract

**Background:**

Diet is a key modifiable risk factor for multiple chronic conditions, including type 2 diabetes (T2D). Consuming a range of foods from the five major food groups is advocated as critical to healthy eating, but the association of diversity across major food groups with T2D is not clear and the relationship of within-food-group diversity is unknown. In addition, there is a growing price gap between more and less healthy foods, which may limit the uptake of varied diets. The current study had two aims: first, to examine the association of reported diversity of intake of food groups as well as their subtypes with risk of developing T2D, and second, to estimate the monetary cost associated with dietary diversity.

**Methods and Findings:**

A prospective study of 23,238 participants in the population-based EPIC-Norfolk cohort completed a baseline Food Frequency Questionnaire in 1993–1997 and were followed up for a median of 10 y. We derived a total diet diversity score and additional scores for diversity within each food group (dairy products, fruits, vegetables, meat and alternatives, and grains). We used multivariable Cox regression analyses for incident diabetes (892 new cases), and multivariable linear regression for diet cost. Greater total diet diversity was associated with 30% lower risk of developing T2D (Hazard ratio [HR] 0.70 [95% CI 0.51 to 0.95]) comparing diets comprising all five food groups to those with three or fewer, adjusting for confounders including obesity and socioeconomic status. In analyses of diversity within each food group, greater diversity in dairy products (HR 0.61 [0.45 to 0.81]), fruits (HR 0.69 [0.52 to 0.90]), and vegetables (HR 0.67 [0.52 to 0.87]) were each associated with lower incident diabetes. The cost of consuming a diet covering all 5 food groups was 18% higher (£4.15/day [4.14 to 4.16]) than one comprising three or fewer groups. Key limitations are the self-reported dietary data and the binary scoring approach whereby some food groups contained both healthy and less healthy food items.

**Conclusions:**

A diet characterized by regular consumption of all five food groups and by greater variety of dairy, fruit, and vegetable subtypes, appears important for a reduced risk of diabetes. However, such a diet is more expensive. Public health efforts to prevent diabetes should include food price policies to promote healthier, more varied diets.

## Abstract 2

Using a large prospective cohort, Annalijn Conklin and colleagues investigate the association between dietary diversity and type 2 diabetes risk.

## Author Summary

**Why was this study done?:**

- Diet is a known modifiable risk factor for chronic diseases, and poor quality diets are linked with risk of type 2 diabetes.
- A varied diet is advocated as being critical to healthy eating, but people can vary in consumption of different food groups, and also of different subtypes within major food groups.

**What did the researchers do and find?:**

- We analysed self-reported diet data and data on new-onset type 2 diabetes diagnosis in middle- and older-aged women and men from the EPIC-Norfolk cohort study.
- Total diet diversity and diversity within major food groups has not previously been studied in relation to health outcomes.
- This large United Kingdom study provides evidence that reported intake of a diet that is diverse in subtypes within the dairy, fruit, and vegetable food groups is independently associated with lower type 2 diabetes risk.
- People who reported consuming all five food groups had a 30% reduced incidence of type 2 diabetes, but the cost of such a diet was 18% higher (£4.15/day [4.14 to 4.16]) than a diet comprising three or fewer food groups.

**What do these findings mean?:**

- Diversity of food groups and subtypes within dairy, fruits, and vegetables is important for chronic disease prevention.
- Health promotion efforts need to incorporate financial strategies to support greater dietary diversity.

## Introduction

Non-communicable diseases present a significant challenge to both high-income and low-income countries, with growing numbers of people experiencing the health and economic burden of one or more chronic conditions [1]. Diet is a key modifiable risk factor for multiple chronic diseases, with poor quality diets being a leading cause of type 2 diabetes (T2D), cardiovascular diseases, hypertension, and certain cancers [2]. It is estimated that diets that do not match nutritional guidelines contribute to 70,000 premature deaths in the United Kingdom [3]. Inadequate consumption of fruits and vegetables in particular is estimated to contribute to 5% of excess mortality globally [2]. Many national and international policies acknowledge the importance of supporting individuals in achieving a healthy balanced diet, and numerous dietary guidelines emphasise the critical role of the consumption of a diet that is varied and includes different foods from different food groups [2,4–7].

Previous aetiological work has tended to examine the association between diet and health by studying individual nutrients, certain food groups, or overall diet quality. Although greater intake of different food subtypes (minor food groups) from each major food group is crucial for nutritional adequacy [8], indices of diet quality rarely include a measure of dietary diversity and none address variety *within* food groups other than for fruits and vegetables [9,10]. Recent prospective studies in the EPIC cohort indicated that consuming a higher number of different items within the fruit (0–58) and/or vegetable (0–59) food groups was associated with a reduced risk of T2D [11] and certain cancers [12,13], independent of known confounders and quantity of intake. Furthermore, specific subtypes of dairy products are also likely to matter for T2D, specifically low-fat fermented items such as yoghurt [14]. Consumption of a higher number of major food groups has been associated with lower all-cause and cause-specific mortality [15,16]. More recently, however, analysis in a multi-ethnic cohort concluded that a higher number of different food items (between 0 and 120) consumed at least twice a week was not associated with incident T2D [17].

It is possible that a diet that is comprised of all five major food groups could still rely on consumption of a narrow range of foods within each food group. In that sense, it would have overall diversity at the major food group consumption level but would not be varied in terms of different subtypes of foods. Therefore, we aimed to investigate how variation between and within each major food group was related to diabetes risk. We hypothesised that greater diversity across major food groups would be associated with lower T2D incidence and that there would be an independent impact of greater diversity of minor food groups within each major group. A secondary aim was to assess the monetary cost associated with dietary diversity, and we expected a greater cost associated with greater diversity.

## Methods

### Ethics Statement

A prescribed informed consent statement was signed by all participants in the EPIC-Norfolk study. The study was approved by the Norwich District Health Authority Ethics Committee.

### Study Population

The EPIC-Norfolk study is a population-based prospective cohort study that has been described in detail elsewhere [18]. In brief, EPIC-Norfolk included 25,639 participants (55% women) aged 40–79 years (99.7% white) who were recruited from age-sex registers of general practices in a geographically circumscribed area in the East of England, and who attended a clinical assessment at cohort entry (1993–1997). Participants were followed up using an 18-mo postal questionnaire, a second clinical assessment (1998–2000), and a second postal questionnaire (2002–2004). We excluded participants with known diabetes at baseline (*n* = 855), unknown diabetes status (*n* = 5), or missing information on potential confounders (*n* = 1,541), providing a final sample of 23,238 individuals for analysis (S1 Fig).

### Case Ascertainment

New T2D cases were ascertained from multiple sources: two follow-up health and lifestyle questionnaires providing self-reported information on doctor-diagnosed diabetes or medications; medications brought to the second clinical exam; and record linkage. Record linkage to external sources included the listing of any EPIC-Norfolk participant in the general practice diabetes register, local hospital diabetes register, hospital admissions data with screening for diabetes-related admissions, and Office of National Statistics mortality data with coding for diabetes. Participants who self-reported a history of diabetes which could not be confirmed against any other sources were not considered as confirmed cases. Follow-up was censored at date of diagnosis of T2D, 31 July 2006, or date of death, whichever came first.

### Dietary Diversity Assessment

A semi-quantitative Food Frequency Questionnaire (FFQ) was used to assess habitual dietary intake at baseline, asking respondents to “estimate average food use during the last year” for 130 of the most commonly consumed food and beverage products. The FFQ provided a standard serving size for each product with nine standard response categories, from never or less than once/month to six or more/day [19]. A separate question was concerned with daily intake of milk, with six possible responses from none to more than one pint.

We used raw frequency data to construct a summary score to assess total diet diversity based on a count of five major food groups used in current food guides for eating well: dairy products, fruits, vegetables, grain/cereal products, and meat and alternatives (protein) [8,20,21]. We also constructed additional scores for dairy diversity (milk, cheese, yoghurt), fruit diversity (vitamin A-rich, citrus and berry, other), vegetable diversity (vitamin A-rich, dark green leafy, starchy tubers, other), “meat and alternatives” diversity (flesh meat—red (including processed), organ meat, flesh meat—poultry, fish and seafood, eggs, legumes/beans and nuts and seeds), and grain diversity (whole grains, non-wholegrains). We assigned individual FFQ items to specific subtypes within each major food group based on previous work [8] and United Nation’s Food and Agriculture Organization food group classification guidance [22] (see S1 Table). Similar to other studies [23], items consumed at least twice per week were considered to constitute habitual intake and counted in the relevant food group. A participant scored zero when they reported intakes of an item to be once a week, 1–3 a month, or never/less than once a month. FFQ responses of one pint (0.5683 L) or more than one pint counted as daily milk intake based on dietary guidelines of 3 cups/d (1.249 imperial pint) [24]. Mixed dishes (e.g., soups, quiche) were separated into main components using codebook description of standard recipes [25], and assigned to relevant food groups and subtypes when ingredients contributed at least 10% to the dish’s total weight or were listed among the top five components. For items with unavailable codebook recipes, we used online lists of ingredients for common brands (e.g., Heinz oxtail soup). Each diversity score increased by one when a different food group was consumed; the score increased regardless of the quantity of an item from a given group or the number of possible items from the same group. We also calculated a composite score for diversity of intake of all food group subtypes (0–18).

Covariables based on completed health and lifestyle questionnaires included education level (four categories), UK Registrar General’s occupational social class (six categories), smoking status (three categories) [26], overall physical activity (four categories), and history of myocardial infarction, stroke, or cancer and family history of diabetes (binary). Waist circumference, height, and weight were measured to standard protocol, and body mass index (BMI) calculated as kg/m^2^.

### Diet Cost Estimation

The monetary cost of the reported diets was estimated by linking food price data for individual foods to the EPIC FFQ’s nutrient composition database as described previously [27]. Retail prices for each of the 289 component food items in the FFQ were obtained by using standardized and published price collection methods [28]. In brief, each food and drink item in the FFQ was priced by using MySupermarket.com, a website for comparing supermarket food prices nationwide in the United Kingdom. For each of the 289 items in the FFQ, we selected the lowest, non-sale price from among the five nationwide retailers on the website at that time (June 2012): Tesco, Sainsbury’s, Asda, Waitrose, and Ocado, which together had a 68% market share at that time [29]. For packaged food (including most fresh produce), we selected the middle size of the range of size options or the larger size if only two options were available. As described previously [28,30], prices were adjusted for preparation losses and cooking fraction to yield an adjusted food price of £/100 g edible portion. The addition of this new variable to the EPIC-Norfolk’s food and nutrient database [31] allowed the derivation of dietary cost for each participant. The variable associated with each individual's diet was cost per day (£/d).

### Data Analysis

Means with standard deviations and frequencies were used to describe the characteristics of the cohort across three levels of the total diet diversity score (≤3, 4, or 5). Covariance matrices were used to assess the strength of relationships between diversity scores. Multivariable Cox regression analyses were used to examine the relationship between each diversity score and the risk of developing T2D. Hazard ratios (HR) and 95% confidence intervals (95% CI) were estimated using a series of models: model 1 adjusted for age, sex, BMI, and total energy intake (Kcal) (*n* = 23,912); model 2 additionally adjusted for lifestyle factors (smoking status, alcohol intake (units/week) and physical activity level) plus family history of diabetes (*n* = 23,705); and model 3 further adjusted for socioeconomic status (education and occupational social class) (*n* = 23,238). Using model 3, the independent relationship of total diet diversity and T2D was then examined by separately including each specific food group diversity score and by including all five specific food group diversity scores. In addition, the independent relationship of each specific food group diversity score with T2D was examined by including (1) the total diet diversity score, (2) the four other specific food group diversity scores, or (3) the total diet diversity score and all other specific food group diversity scores.

Sensitivity analyses included the total quantity of intake of all items from the relevant food group in model 3 to control for the relationship between the diversity of food groups and the number of foods reported, which is independently associated with nutrient adequacy [32]. Waist circumference, as a marker of central adiposity, was also included in model 3, as it may be an independent risk factor of cardio-metabolic conditions [33]. Vegetable diversity was re-examined after excluding all potato items and, alternatively, restricting to baked and boiled potatoes given the high consumption in the UK of fried potato products, which would contribute to higher fat and energy intakes. Analyses were also repeated after additionally excluding participants with self-reported chronic conditions. We also undertook a sensitivity analysis in the sub-sample of EPIC-Norfolk (*n* = 10,787) in whom HbA_1c_ was measured at baseline to exclude individuals (*n* = 262) who had a baseline HbA_1c_ ≥6.5% (or ≥48 mmol/mol), which is indicative of prevalent but undiagnosed diabetes.

Multivariable linear regression was used to assess cross-sectional associations at baseline between each diversity score and diet cost, adjusting for age, sex, and total energy intake (*n* = 23,238). We used regression coefficients for post-estimation calculation of adjusted means (95% CI). Statistical analyses were conducted using Stata version 13.1.

## Results

The average duration of follow up was 10 (±1.5) y, and we identified 892 new cases of T2D over 245,045 person-years of follow up. On average, participants reported consuming 4.7 (0.6) major food groups at least twice or more per week. Very few participants reported consuming foods from two groups (0.45%), one group (0.07%), or none (0.01%); while most reported consuming four (21.29%) or five groups (74.43%) and some consumed only three groups (3.75%). Within the specific food groups, there was more evidence of heterogeneity in reported diets between individuals. A diversity score of zero was observed in 13.4% of participants for dairy products, 7.8% for fruits, and 8.1% for meat (and alternatives). For participants who scored three for total diet diversity, we found that 80% scored zero for dairy, 62% for fruit, 10% for vegetables, 55% for meat, and 9% for grain. And among participants who scored four for total diet diversity, there were 47% scoring zero for dairy, 24% for fruit, 1% for vegetables, 27% for meat and 1% for grain.

Total diet diversity was positively correlated with diversity within each specific food group: dairy (r = 0.52), fruits (r = 0.42), vegetables (r = 0.28), meat and alternatives (r = 0.35), and grains (r = 0.21). The specific food group diversity scores were not correlated with each other, except for the scores for diversity in fruits and vegetables (r = 0.23), and vegetables and meat and alternatives (r = 0.25). Table 1 shows that participants who reported regular consumption of a diet with greater total diet diversity had more favourable socioeconomic and lifestyle profiles.

**Table 1:** Baseline characteristics of total diet diversity in participants in the EPIC-Norfolk study.

	Total diet diversity score^1^			
	≤3	4	5	*p*-value
*n*	986	4,920	17,332	
Age at recruitment	57.8 (9.2)	58.3 (9.3)	59.2 (9.2)	<0.001
Women, *n* (%)	515 (52)	2,587 (53)	9,631 (56)	<0.001
Education level, *n* (%)				<0.001
No qualification (<11 y)	412 (42)	1,873 (38)	6,013 (35)	
O level (11 y)	95 (10)	519 (11)	1,817 (10)	
A level (≥13 y)	378 (38)	1,938 (39)	7,178 (41)	
Degree (≥16 y)	101 (10)	590 (12)	2,324 (13)	
Occupational social class, *n* (%)				<0.001
Unskilled	48 (5)	196 (4)	551 (3)	
Partly skilled	143 (15)	699 (14)	2,199 (13)	
Skilled–manual	251 (25)	1,201 (24)	3,882 (22)	
Skilled–non manual	153 (16)	787 (16)	2,919 (17)	
Managerial & Technical	340 (34)	1,708 (35)	6,538 (38)	
Professional	51 (5)	329 (7)	1,243 (7)	
Physical activity, *n* (%)				<0.001
Inactive	343 (35)	1,551 (32)	4,922 (28)	
Moderately inactive	277 (28)	1,410 (29)	5,051 (29)	
Moderately active	216 (22)	1,102 (22)	4,056 (23)	
Active	150 (15)	857 (17)	3,303 (19)	
Smoking status, *n* (%)				<0.001
Current smoker	222 (23)	805 (16)	1,686 (10)	
Former smoker	385 (39)	1,964 (40)	7,386 (43)	
Never smoker	379 (38)	2,151 (44)	8,260 (48)	
BMI (Kg/m^2^)	26.1 (3.9)	26.2 (3.9)	26.3 (3.8)	0.01
Waist circumference (cm)	88.2 (12.3)	88.0 (12.3)	88.0 (12.2)	0.546
Total energy intake (kcal/d)	1,543 (500)	1,816 (519)	2,147 (590)	<0.001
Total alcohol intake (g/d)	136 (268)	144 (275)	125 (224)	0.004

^1^ Number of major food groups (0–5) consumed at least twice a week.

*P*-values are from the test for trend for continuous variables and the χ^2^ test for categorical variables.

As shown in Table 2, the total diet diversity score and the specific food group diversity scores for dairy products, fruits, and vegetables were each inversely associated with risk of developing T2D (Model 1). Participants who reported meeting the recommendation to consume foods from all five food groups had a 30% lower incidence of T2D (HR 0.70 [0.51, 0.95]), but those consuming only four major food groups did not have a lower risk (HR 0.85 [0.62, 1.18]) compared to those reporting intakes of three or fewer food groups. Similarly, those participants who reported the greatest level of diversity of consumption of dairy products, fruits, or vegetables had a 38% (HR 0.62 [0.47, 0.83]), 35% (HR 0.65 [0.50, 0.84]), and 33% (HR 0.67 [0.52, 0.86]), respectively, lower risk of T2D compared to the individuals with the least variation of subtypes within a specific food group. In the case of these three specific food groups, there was a significant linear trend with the risk of developing diabetes being inversely related to the degree of food group diversity. There was no association with diversity within the meat or grain food groups. Adjustment for family history and lifestyle factors (Model 2) and additionally for socioeconomic status (Model 3) did not appreciably alter the HRs. We also observed a strong inverse association between the summary score for diversity of all food group subtypes and risk of developing type 2 diabetes (*p* for trend <0.01) (S2 Table).

**Table 2:** Adjusted hazard ratios (95% CI) of incident diabetes for total diet diversity and for diversity within each major food group in the EPIC-Norfolk study.

Score	*n* of food groups^1^	Cases/total	*n* of events (rate per 100,000 person years)	Model 1		Model 2		Model 3	
				HR	*95% CI*	HR	*95% CI*	HR	*95% CI*
Total diet diversity	0–3	46/1,028	453	1		1		1	
	4	204/5,104	405	0.84	*0*.*61, 1*.*17*	0.83	*0*.*60, 1*.*15*	0.85	*0*.*62, 1*.*18*
	5	600/17,838	340	0.70*	*0*.*51, 0*.*95*	0.68*	*0*.*50, 0*.*93*	0.70*	*0*.*51, 0*.*95*
			*p*-trend	*0*.*0223*		*0*.*0163*		*0*.*0236*	
Dairy diversity	0	144/3,219	455	1		1		1	
	1	306/8,829	350	0.76**	*0*.*62, 0*.*93*	0.73**	*0*.*60, 0*.*89*	0.72**	*0*.*59, 0*.*89*
	2	321/9,145	355	0.76**	*0*.*62, 0*.*94*	0.76**	*0*.*62, 0*.*93*	0.75**	*0*.*61, 0*.*93*
	3	79/2,777	286	0.62**	*0*.*47, 0*.*83*	0.62**	*0*.*46, 0*.*83*	0.61**	*0*.*45, 0*.*81*
			*p*-trend	*0*.*0017*		*0*.*0021*		*0*.*0015*	
Fruit diversity	0	83/1,861	450	1		1		1	
	1	168/4,008	423	0.87	*0*.*67, 1*.*13*	0.86	*0*.*66, 1*.*12*	0.88	*0*.*67, 1*.*15*
	2	396/10,570	377	0.79	*0*.*62, 1*.*00*	0.78*	*0*.*61, 0*.*99*	0.81	*0*.*63, 1*.*04*
	3	203/7,531	274	0.65**	*0*.*50, 0*.*84*	0.64***	*0*.*49, 0*.*84*	0.69**	*0*.*52, 0*.*90*
			*p*-trend	*0*.*0006*		*0*.*0008*		*0*.*0053*	
Vegetable diversity	0–1	79/1,511	530	1		1		1	
	2	174/4,414	397	0.78	*0*.*60, 1*.*03*	0.79	*0*.*60, 1*.*03*	0.79	*0*.*60, 1*.*03*
	3	289/8,484	343	0.69**	*0*.*54, 0*.*89*	0.69**	*0*.*53, 0*.*89*	0.69**	*0*.*54, 0*.*90*
	4	308/9,561	328	0.67**	*0*.*52, 0*.*86*	0.66**	*0*.*51, 0*.*85*	0.67**	*0*.*52, 0*.*87*
			*p*-trend	*0*.*0009*		*0*.*0006*		*0*.*0013*	
Meat diversity	0	64/1,941	334	1		1		1	
	1	192/5,491	354	1.08	*0*.*81, 1*.*43*	1.06	*0*.*80, 1*.*42*	1.04	*0*.*78, 1*.*39*
	2	256/7,189	359	1.08	*0*.*82, 1*.*43*	1.08	*0*.*82, 1*.*43*	1.02	*0*.*77, 1*.*35*
	3	191/5,945	325	0.93	*0*.*69, 1*.*25*	0.93	*0*.*69, 1*.*25*	0.90	*0*.*67, 1*.*21*
	4	116/2,731	432	1.16	*0*.*84, 1*.*59*	1.17	*0*.*85, 1*.*61*	1.16	*0*.*84, 1*.*60*
	5–6	31/673	470	1.09	*0*.*70, 1*.*71*	1.19	*0*.*76, 1*.*86*	1.15	*0*.*73, 1*.*81*
			*p*-trend	*0*.*6766*		*0*.*4275*		*0*.*4807*	
Grain diversity	0–1	162/4,016	407	1		1		1	
	2	688/19,954	349	0.95	*0*.*80, 1*.*13*	0.95	*0*.*80, 1*.*14*	0.96	*0*.*81, 1*.*15*
			*p*-trend	*0*.*5895*		*0*.*5978*		*0*.*6803*	

Model 1 (*n* = 23,912) was adjusted for age, sex, BMI, and total energy intake (kcal/d)

Model 2 (*n* = 23,705) as model 1 plus: smoking status, total alcohol intake, physical activity level and family history of diabetes

Model 3 (*n* = 23,238) as model 2 plus: education and occupational social class

*p < 0.05;

**p < 0.01;

***p < 0.001

^1^ Diversity scores were based on the number of different major food groups, or the number of minor food groups (subtypes) within a major group.

After additionally mutually adjusting for all diversity scores within specific food groups, the inverse association of total diet diversity with diabetes risk was attenuated and became non-significant (*p* = 0.47) (Table 3, Model 6). In analyses adjusting for the association of other specific food group diversity scores, the inverse association of dairy, fruit, and vegetable diversity with T2D remained statistically significant (Table 4, Model 2). However, after accounting for total diet diversity and all other specific food group diversity scores, only dairy and vegetable diversity were significantly independently associated with diabetes risk (Table 4, Model 3).

**Table 3:** Adjusted hazard ratios (95% CI) of incident diabetes for total diet diversity in the EPIC-Norfolk study, independent of diversity within specific food groups.

Score	*n* of food groups	Base: covariable- and SES-adjusted		Model 1: base + dairy diversity		Model 2: base + fruit diversity		Model 3: base + vegetable diversity		Model 4: base + meat & alternative diversity		Model 5: base + grain diversity		Model 6: base + all 5 within-group diversity scores	
		HR	*95% CI*	HR	*95% CI*	HR	*95% CI*	HR	*95% CI*	HR	*95% CI*	HR	*95% CI*	HR	*95% CI*
Total diet diversity	0–3	1		1		1		1		1		1		1	
	4	0.85	*0*.*62*, *1*.*18*	0.88	*0*.*64*, *1*.*23*	0.91	*0*.*65*, *1*.*27*	0.90	*0*.*65*, *1*.*25*	0.84	*0*.*60*, *1*.*16*	0.86	*0*.*62*, *1*.*18*	0.96	*0*.*68, 1*.*35*
	5	0.70*	*0*.*51*, *0*.*95*	0.77	*0*.*55*, *1*.*07*	0.78	*0*.*56*, *1*.*09*	0.70	*0*.*54*, *1*.*03*	0.67*	*0*.*48*, *0*.*92*	0.70*	*0*.*51*, *0*.*95*	0.87	*0*.*60, 1*.*27*

Base model adjusted for age, sex, BMI, total energy intake (kcal/d), smoking status, total alcohol intake, physical activity level and family history of diabetes and SES. Separate models additionally adjusted for diversity of dairy product subtypes (Model 1), fruit subtypes (Model 2), vegetable subtypes (Model 3), meat and alternative subtypes (Model 4), grain subtypes (Model 5), or all five within-group diversity scores (*n* = 23,238).

*p < 0.05;

**p < 0.01;

***p < 0.001

**Table 4:** Adjusted hazard ratios (95% CI) of incident diabetes for diversity of dairy products, fruits, vegetables, grains, and meat products in the EPIC-Norfolk study, independent of total diet diversity and diversity within other food groups.

Score	*n* of food groups^1^	Model 1: + total diet diversity score		Model 2: + scores for diversity within other food groups		Model 3: Model 1 + Model 2	
		HR	*95% CI*	HR	*95% CI*	HR	*95% CI*
Dairy diversity	0	1		1		1	
	1	0.80	*0*.*61*, *1*.*04*	0.73**	*0*.*60, 0*.*90*	0.72*	*0*.*53*, *0*.*96*
	2	0.83	*0*.*63*, *1*.*10*	0.78*	*0*.*63, 0*.*97*	0.76	*0*.*56*, *1*.*03*
	3	0.67*	*0*.*47*, *0*.*95*	0.64**	*0*.*48, 0*.*86*	0.62**	*0*.*43*, *0*.*90*
Fruit diversity	0	1		1		1	
	1	1.05	*0*.*77*, *1*.*43*	0.91	*0*.*70, 1*.*19*	1.02	*0*.*74*, *1*.*42*
	2	0.97	*0*.*73*, *1*.*31*	0.86	*0*.*67, 1*.*10*	0.96	*0*.*71*, *1*.*31*
	3	0.84	*0*.*61*, *1*.*15*	0.75*	*0*.*57, 0*.*99*	0.84	*0*.*60*, *1*.*17*
Vegetable diversity	0–1	1		1		1	
	2	0.83	*0*.*63*, *1*.*10*	0.81	*0*.*61, 1*.*06*	0.82	*0*.*62*, *1*.*08*
	3	0.74*	*0*.*57*, *0*.*97*	0.72*	*0*.*55, 0*.*94*	0.73*	*0*.*56*, *0*.*96*
	4	0.73*	*0*.*56*, *0*.*95*	0.71*	*0*.*54, 0*.*93*	0.72*	*0*.*55*, *0*.*95*
Meat diversity	0	1		1		1	
	1	1.35	*0*.*98*, *1*.*85*	1.09	*0*.*82, 1*.*46*	1.22	*0*.*87*, *1*.*72*
	2	1.32	*0*.*96*, *1*.*81*	1.11	*0*.*83, 1*.*47*	1.24	*0*.*88*, *1*.*73*
	3	1.17	*0*.*84*, *1*.*62*	0.99	*0*.*73, 1*.*33*	1.10	*0*.*77*, *1*.*56*
	4	1.51*	*1*.*06*, *2*.*14*	1.30	*0*.*94, 1*.*80*	1.45	*1*.*00*, *2*.*10*
	5–6	1.49	*0*.*93*, *2*.*41*	1.30	*0*.*82, 2*.*06*	1.44	*0*.*88*, *2*.*36*
Grain diversity	0–1	1		1		1	
	2	0.99	*0*.*83*, *1*.*18*	1.02	*0*.*85, 1*.*22*	1.02	*0*.*85*, *1*.*22*

Hazard ratios were adjusted for key covariables (age, sex, BMI, energy, lifestyle factors, family history, and SES) as well as for total diet diversity (Model 1), or for diversity within the other four major food groups (Model 2), or both (Model 3) (*n* = 23,238)

*p < 0.05;

**p < 0.01;

***p < 0.001

^1^ Diversity scores were based on the number of different major food groups, or the number of minor food groups (subtypes) within a major group.

Inclusion of total quantity of all items from a given food group attenuated results for total diet diversity and dairy diversity, although inverse associations were amplified for vegetable diversity and unaffected for fruit diversity. Results were unaffected in sensitivity analyses after including waist circumference or excluding participants with self-reported chronic conditions. After excluding participants with a baseline level of HbA_1c_ ≥ 6.5%, greatest fruit diversity and vegetable diversity showed stronger inverse associations with T2D (HR 0.42 [0.23, 0.75] and HR 0.56 [0.32, 0.97], respectively) as did total diet diversity (HR 0.53 [0.28, 1.00]) (S3 Table). Finally, inverse associations were stronger for total diet diversity and similar for vegetable diversity when we counted only baked and boiled potatoes, or did not count potato items consumed at least twice a week (S4 Table).

The adjusted mean diet cost was 18% higher for participants consuming all five major food groups (£4.15/day [4.14 to 4.16]), and 7% for four major food groups (£3.85/day [3.83 to 3.88]), compared to those reporting limited diversity (£3.53/day [3.48 to 3.59]) (Table 5 and S2 Fig). The comparison for the costs of diversity within the specific food groups suggested differences between extreme categories of 7% (£0.29/day) for dairy diversity (4.25 [4.22, 4.29] versus 3.96 [3.93, 3.99]), 22% (£0.81/day) for fruit diversity (4.43 [4.41, 4.45] versus 3.63 [3.59, 3.67]), 30% (£1.01/day) for vegetable diversity (4.40 [4.38, 4.42] versus 3.39 [3.35, 3.44]), 42% (£1.47/day) for meat (and alternatives) diversity (4.93 [4.87, 5.00] versus 3.48 [3.44, 3.51]), and -1% (£0.06/day) for grain diversity (4.05 [4.04, 4.06] versus 4.11 [4.08, 4.14]) (Table 5 and S2 Fig). The summary score for diversity of all food group subtypes was also associated with a significant added diet cost (*p* for trend < 0.001) (Table 5).

**Table 5:** Adjusted mean daily diet cost (95% CI) of total diet diversity and of diversity within each major food group in the EPIC-Norfolk study.

Score	*n* of food groups^1^	Mean (£/d)	*95% CI*
Total diet diversity	0–3	3.53	*3*.*48, 3*.*59*
	4	3.85	*3*.*83, 3*.*88*
	5	4.15	*4*.*14, 4*.*16*
	*p*-trend	*<0*.*001*	
Dairy diversity	0	3.96	*3*.*93, 3*.*99*
	1	4.00	*3*.*98, 4*.*02*
	2	4.10	*4*.*08, 4*.*12*
	3	4.25	*4*.*22, 4*.*29*
	*p*-trend	*<0*.*001*	
Fruit diversity	0	3.63	*3*.*59, 3*.*67*
	1	3.72	*3*.*69, 3*.*74*
	2	4.00	*3*.*99, 4*.*02*
	3	4.43	*4*.*41, 4*.*45*
	*p*-trend	*<0*.*001*	
Vegetable diversity	0–1	3.39	*3*.*35, 3*.*44*
	2	3.71	*3*.*68, 3*.*73*
	3	3.98	*3*.*96, 4*.*00*
	4	4.40	*4*.*38, 4*.*42*
	*p*-trend	*<0*.*001*	
Meat diversity	0	3.48	*3*.*44, 3*.*51*
	1	3.72	*3*.*69, 3*.*74*
	2	4.02	*4*.*00, 4*.*04*
	3	4.29	*4*.*27, 4*.*31*
	4	4.56	*4*.*53, 4*.*59*
	5–6	4.93	*4*.*87, 5*.*00*
	*p*-trend	*<0*.*001*	
Grain diversity	0–1	4.11	*4*.*08, 4*.*14*
	2	4.05	*4*.*04, 4*.*06*
	*p-*trend	*<0*.*001*	

Means obtained by multivariable linear regression analysis adjusted for sex, age, and total energy intake (kcal/d) (*n* = 23,238).

^1^ Diversity scores were based on the number of different major food groups, or the number of minor food groups (subtypes) within a major group.

## Discussion

This prospective population-based cohort study of 23,238 British adults suggested that individuals who report regular weekly consumption of all five major food groups subsequently had a lower risk of developing type 2 diabetes as did people who consumed diets that were rich in variability within the dairy, fruit, and vegetable food groups. The association of total diet diversity was attenuated after accounting for diversity within the five food groups. However, greater diversity within dairy, fruit, and vegetable food groups remained predictive of diabetes, independent of diversity in each of the other food groups. The cost of a diet that was varied was significantly higher than the cost of one that was the least diverse.

Previous epidemiological studies show that several diet quality indices are associated with 9%–13% reduced risk of T2D [9,10]. However, these studies do not separately examine the role of dietary diversity in relation to T2D. Variety of foods is only considered as a component of a few diet quality indices (e.g., Healthy Eating Index and Dietary Guidelines Index) [9,10]. Studies using diet diaries have shown the risk of T2D is lower by 14%–21% in people reporting higher vegetable intake, particularly green leafy vegetables [11,34], and by 15%–28% with greater reported dairy product intake, specifically yoghurt consumption [14,35]. Previous studies have also examined the broader health impact of total diet diversity, showing higher risk of mortality in people who reported consuming diets with only two food groups or fewer per week when measured by 24 h recall [15,16]. To date, the only published study that examined diversity within specific food groups (fruits and vegetables), showed that people who reported consuming 12 different fruit and vegetable items per week had a 39% lower risk of developing T2D [11]. Despite common advice to consume a varied diet [2,5,7], we are not aware of studies investigating how the number of different food groups and different subtypes within each food group included in a diet are associated with risk of diabetes. Our findings suggest that individuals who meet the recommendation to consume a healthy diet with food items from each of the five food groups had a reduced risk of developing T2D. More notably, our results further showed that people reporting regular consumption of the full range of food subtypes within dairy, fruit, and vegetable food groups also had a reduced risk of T2D.

The biological pathways linking the inverse associations of total diet diversity and diversity within three specific food groups with T2D risk are unclear. A recent study using FFQ data in older adults reports that greater diversity of foods consumed was significantly positively correlated with a more diverse intestinal microbiota, suggesting that dietary diversity influences microbiota composition [36]. Complementary experiments in that study further showed that dietary changes toward lower diversity resulted in losses in the range of different intestinal microbiota and that reduced microbiota diversity was associated with poorer health outcomes [36]. Greater within-group diversity may also have a specific role for health by providing a balance of the multitude of micronutrients, dietary fibre, and other bioactive compounds necessary for maintaining physical functioning [37]. The particular benefits of fruit and vegetable diversity may derive from the inclusion of phytochemicals that are more specific to certain subgroups that individuals with more varied intakes might consume preferentially [38]. For example, greater vegetable diversity may provide individuals with specific subgroups that contain high concentrations of flavonoids and carotenoids, which have known health benefits [39].

While diverse diets may be healthier, they are also more costly [40–42]. Others have reported a difference of 12% in total weekly food expenditure when comparing top and bottom ranges of food variety [43,44]. In the current study, the adjusted mean cost of the whole diet was 18% higher for participants consuming all five food groups compared to those consuming only three or fewer groups. In light of global 5-a-day campaigns emphasising fruit and vegetable variety, it is important for public health efforts to acknowledge that the adoption of diets including all vegetable and all fruit subtypes may be substantially more costly for consumers and may especially exacerbate existing socioeconomic inequalities in diet. Others also note the higher cost of better quality diets [28,45]. Modelling evidence indicates that combining food taxes and subsidies as a multifaceted policy intervention could best support individuals in making healthy food choices so as to prevent chronic conditions and to help reduce health disparities [46,47]. Given the rising price of healthy food groups, there is a need for a comprehensive food pricing strategy to target the increase in the diversity of foods individuals consume, particularly within fruits and vegetables. Further work should investigate how to develop and implement such a policy approach and to evaluate the impact on equity.

The strengths and weaknesses of this study deserve attention. Strengths include a large sample size, prospective study design, thorough assessment of new cases of T2D with self-report information supplemented by external sources, use of established classification of food groups, and comprehensive information on covariables, thereby minimising sources of bias and confounding. In particular, we examined the exposure to different subtypes within each major food group using two approaches (separate within-group scores and a composite score of all food subtypes). The greater magnitude of effect on diabetes incidence and more pronounced diet cost using the composite score further corroborates the primary findings. Another strength of our study was the availability in a subgroup of HbA_1c_ data at baseline, allowing us to confirm that our findings were unaffected by undiagnosed cases of T2D at baseline. However, some potential limitations merit discussion. First, as an observational study, results may be limited by residual confounding or confounding by unmeasured factors. Second, dietary data were based on self-report from FFQ and therefore may be prone to error and bias [19]. In particular, participants who reported diets with limited variety of food groups may have poor completion of the FFQ. Nonetheless, we took an over-inclusive scoring approach, which likely captured diets that had lower levels of diversity. Moreover, FFQ data are suitable for ranking individuals according to habitual intakes and our scoring approach using frequency information avoided the many assumptions used to estimate absolute intakes [19]. However, the diversity scores were limited by the fact that they were based on a simple yes/no for consumption at least twice a week, regardless of the amount consumed, the number of items consumed within a given food group, or the potential healthfulness of an item (e.g., whole-fat versus low-fat milk, lean meats versus red and processed meats, fried fish versus baked fish). In addition, our study did not account for changes in diet diversity and/or changes in other lifestyle factors over follow-up, and our price data were from 2012 because information was not available retrospectively for study baseline. Finally, the EPIC-Norfolk data provides strong external validity and generalisability only to other predominantly European-descended and middle-aged populations.

### Conclusion

This large epidemiological study in a population-based cohort is the first to report an association of total diet diversity and diversity within specific food groups with lower risk of diabetes. These findings support current public health recommendations encouraging consumption of all major food groups and also of different types of fruits, vegetables, and dairy products as part of a regular balanced diet. However, the additional cost of greater diversity deserves attention toward a comprehensive food pricing strategy. Future work should investigate how to develop and implement such a policy approach, including the consideration of financial incentives to actively support lower-income groups in achieving a healthy, mixed diet.

## Supporting Information

S1 Fig Flow diagram for sample restriction in the EPIC-Norfolk cohort for analyses reported in this study. (TIF)

S2 Fig Adjusted mean daily diet cost associated with total diet diversity and with diversity within each major food group in the EPIC-Norfolk study. Multivariable linear regression adjusted for sex, age, and total energy intake (kcal/d) (*n* = 23,238, *p*-trend < 0.001). Diversity scores were based on the number of different major food groups or the number of minor food groups (subtypes) consumed within a major group. Data is presented in Table 5.
(DOCX)

S1 Table Classification of food items in the EPIC-Norfolk study Food Frequency Questionnaire into five major food groups and their subtypes. ^1^ Based on reference [8] and Annex 2 on food group assignment in [22]. ^2^ Mixed dishes were separated into component ingredients using standardized recipes (McCance and Widdowson’s *The Composition of Foods*, 6th edition) [25] and assigned to food groups when ingredients in that group comprised at least 10% of the dish.
(DOCX)

S2 Table Adjusted hazard ratios (95% CI) of incident diabetes in relation to quintiles of a summary score for diversity of all food group subtypes in the EPIC-Norfolk study. Model 1 (*n* = 23,912) was adjusted for age, sex, BMI, and total energy intake (kcal/d). Model 2 (*n* = 23,705) as Model 1 plus the following: smoking status, total alcohol intake, physical activity level, and family history of diabetes. Model 3 (*n* = 23,238) as Model 2 plus the following: education and occupational social class. * *p* < 0.05; ** *p* < 0.01; *** *p* < 0.001. ^1^ All food subtypes within each food group were summed together to create a composite continuous score ranging from zero to 18 subtypes across five major food groups.
(DOCX)

S3 Table Sensitivity analyses of incident diabetes in relation to total diet diversity and diversity within each major food group in the EPIC-Norfolk study. Model A adjusted for all covariables and total quantity of the dependent variable examined (*n* = 23,105). Model B adjusted for all covariables and waist circumference (*n* = 23,222). Model C adjusted for all covariables after excluding participants with HbA_1c_ ≥ 6.5% (*n* = 9,914). Model D was adjusted for all covariables after excluding participants with self-reported health conditions (*n* = 21,110). * *p* < 0.05; ** *p* < 0.01; *** *p* < 0.001.
(DOCX)

S4 Table Sensitivity analyses of incident diabetes in relation to diversity scores excluding all potato items or including only boiled and baked potato items in the EPIC-Norfolk study. Model A adjusted for all covariables using a revised score for vegetable diversity and total diet diversity, without counting any potato items reported by EPIC participants. Model B adjusted for all covariables using a revised score for vegetable diversity and total diet diversity, counting only baked or boiled potato items reported by EPIC participants. *n* = 23,238. * *p* < 0.05; ** *p* < 0.01; *** *p* < 0.001. ^1^ The revised score for diversity of vegetable subtypes used the score of zero as the reference group since there were sufficient numbers at that level (*n* = 1,194, 5%). ^2^ The revised score for diversity of vegetable subtypes used the combined scores of zero and one as the reference group due to low numbers at the zero level (*n* = 225, 0.94%)
(DOCX)

S5 Table Adjusted mean daily diet cost (95% CI) associated with of a summary score for diversity of all food group subtypes in the EPIC-Norfolk study. Means obtained by multivariable linear regression analysis adjusted for sex, age, and total energy intake (kcal/d) (*n* = 23,238). ^1^ All food subtypes within each food group were summed together to create a composite continuous score ranging from zero to 18 subtypes across five major food groups.
(DOCX)

S1 Protocol The prospective study protocol developed January 21, 2015. (DOCX)

S1 STROBE Checklist Completed strengthening the reporting of observational studies in epidemiology (STROBE) checklist. (DOCX)

## Abbreviations

BMI: body mass index
FFQ: Food Frequency Questionnaire
HR: Hazard ratio
T2D: type 2 diabetes
