# Correction: Dietary Diversity, Diet Cost, and Incidence of Type 2 Diabetes in the United Kingdom: A Prospective Cohort Study

**DOI:** 10.1371/journal.pmed.1002123

**Published:** 2016-08-19

**Authors:** The PLOS Medicine Staff

## Abstract

Corrected publisher error in data availability statement.

The information in the Data Availability section is incorrect. The correct data availability information is as follows: Data from the EPIC Norfolk Study cannot be deposited publicly due to ethics and consent issues. The EPIC-Norfolk data access committee can be contacted at epic@srl.cam.ac.uk.

The publisher apologizes for the error.
