# Supplementary figures and images for: Dietary Diversity, Diet Cost, and Incidence of Type 2 Diabetes in the United Kingdom: A Prospective Cohort Study
Source: PLoS Med. 2016 Jul 19;13(7):e1002085. doi: 10.1371/journal.pmed.1002085 (PMC4951147; doi:10.1371/journal.pmed.1002085)

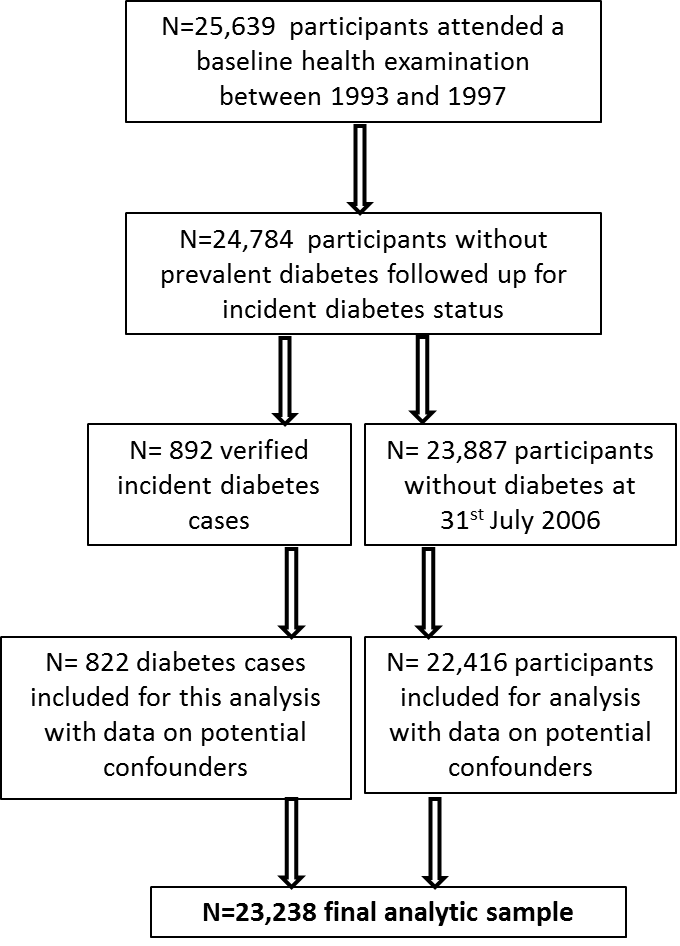

Supplement: S1 Fig — (TIF) [file pmed.1002085.s001.tif]

**A**


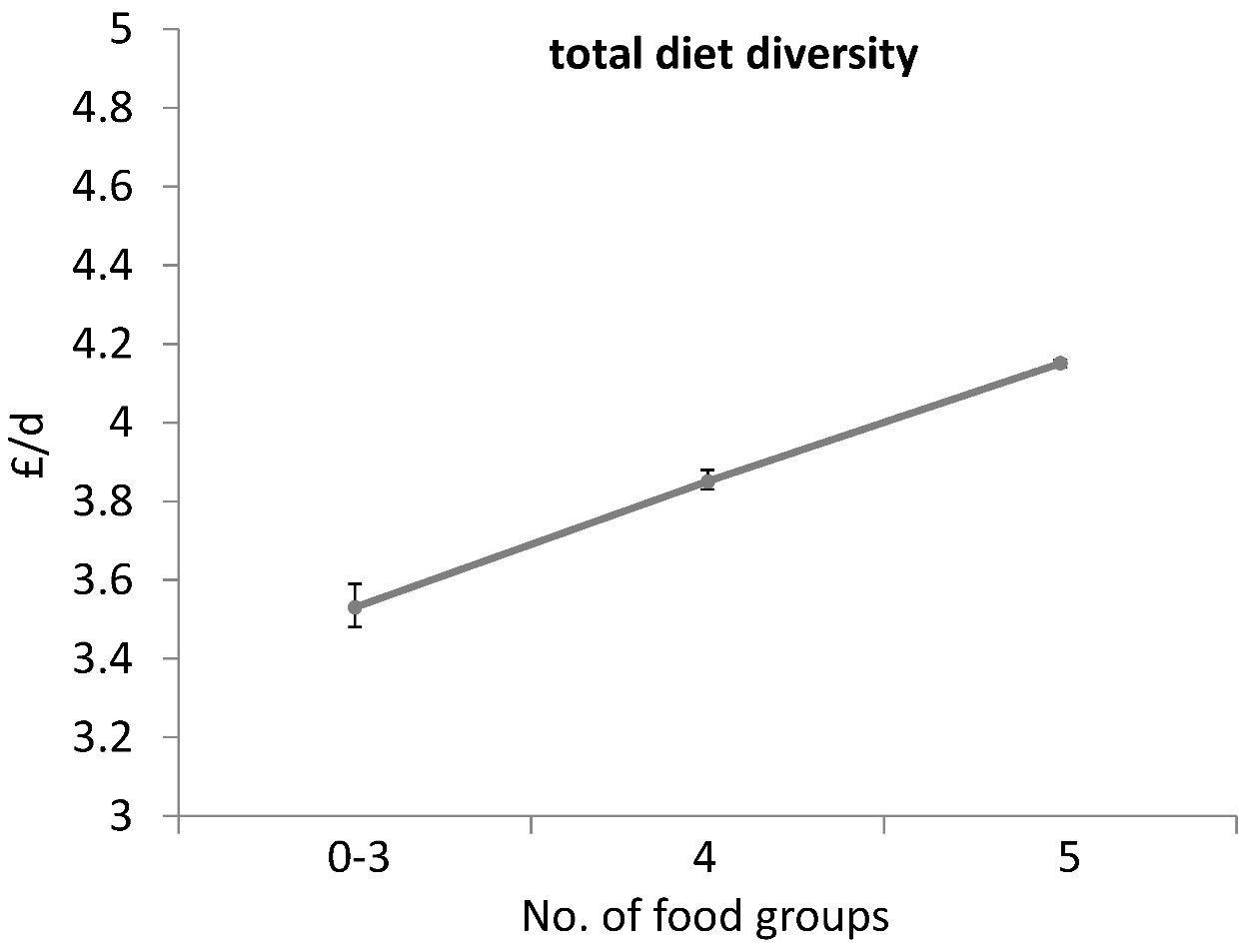


**B**


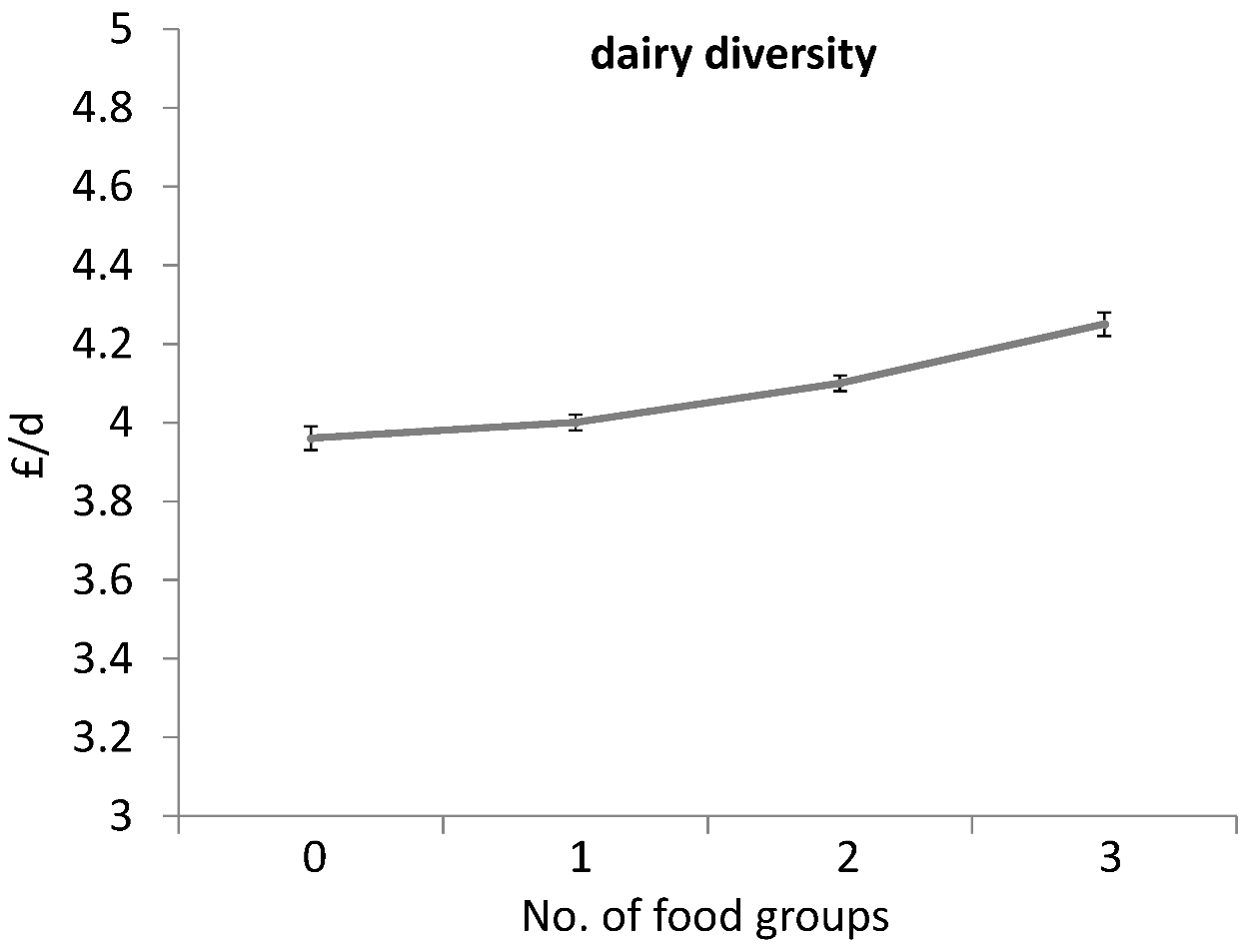


**C**


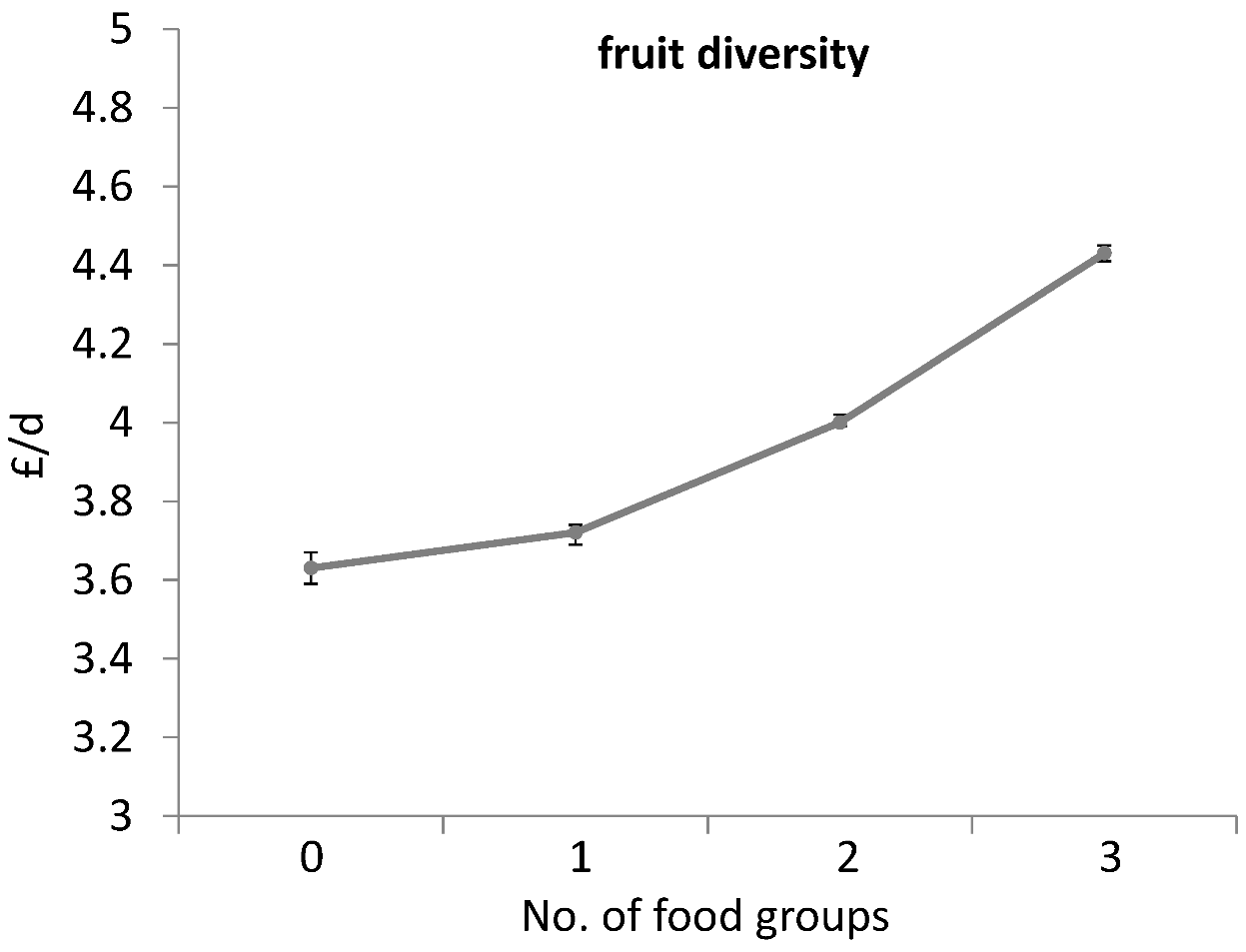


**D**


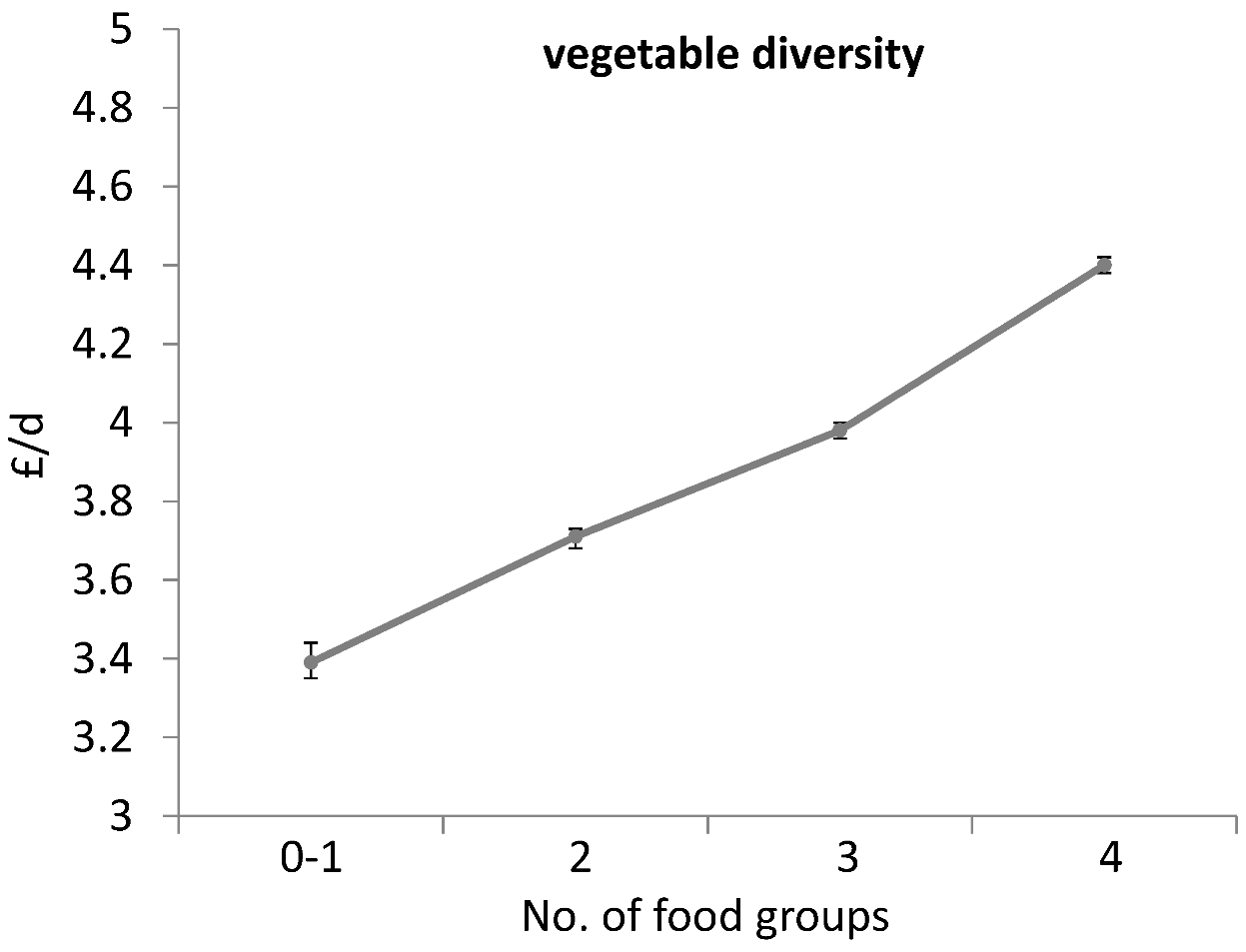


**E**


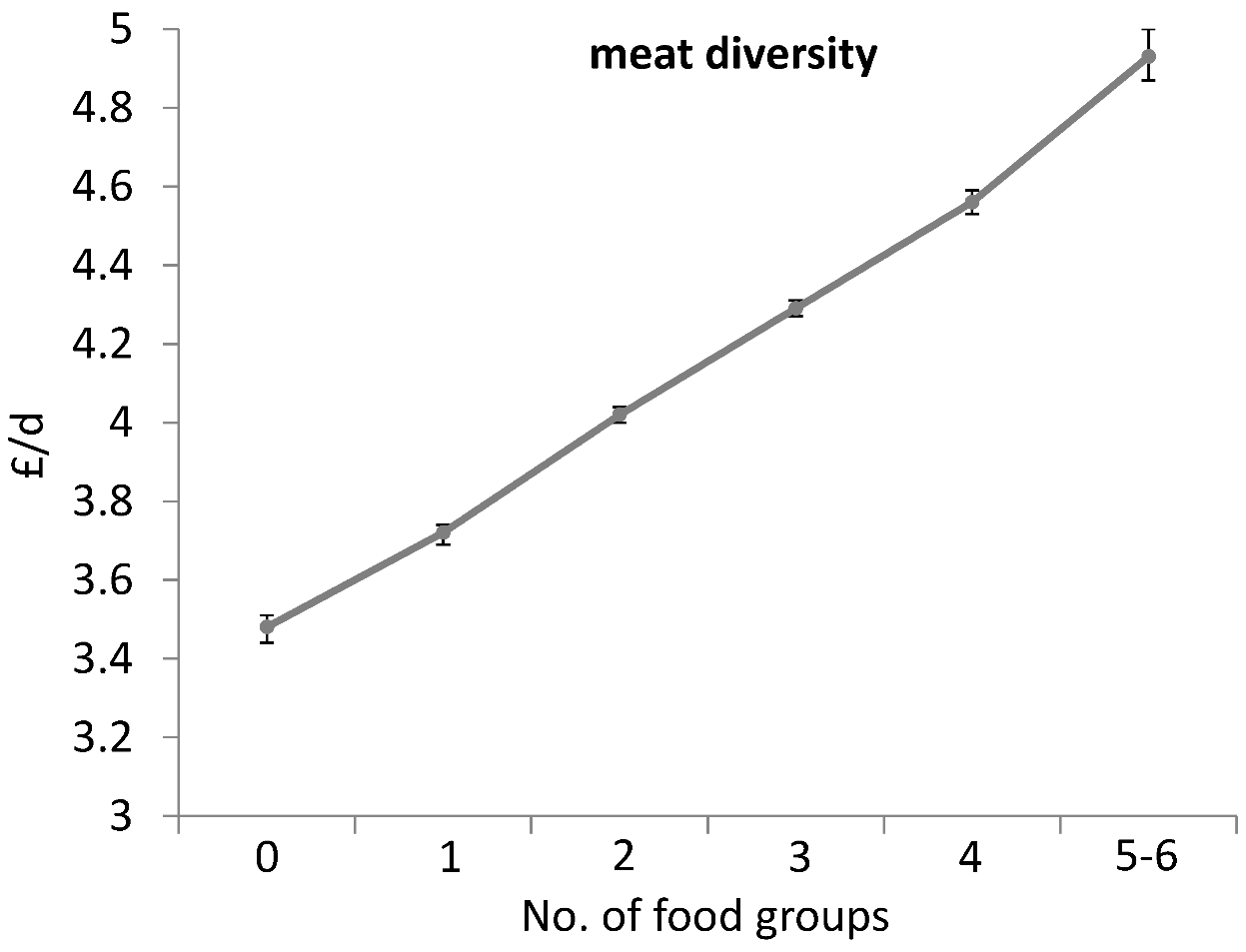


**F**

**
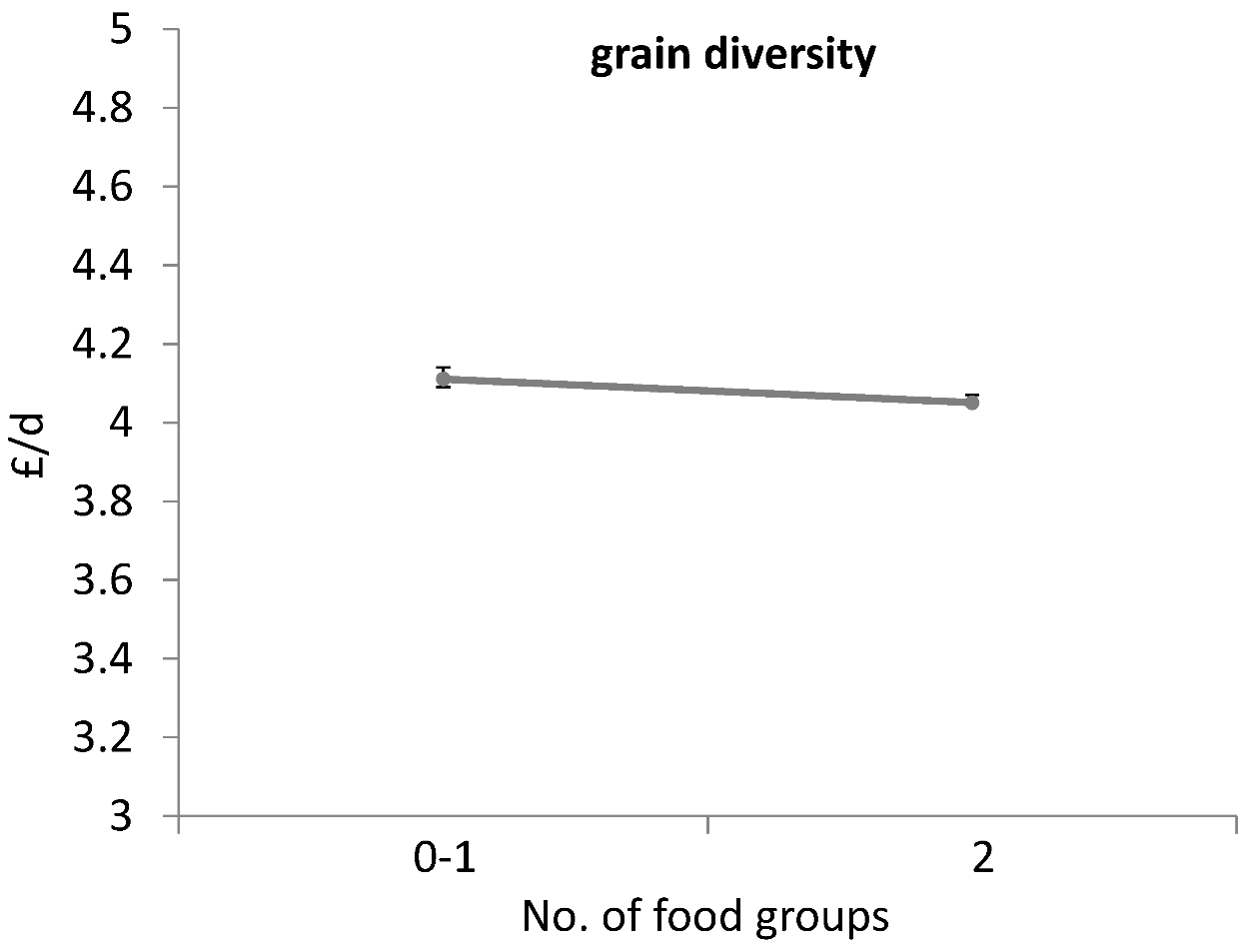
**

Supplement: S2 Fig — Multivariable linear regression adjusted for sex, age, and total energy intake (kcal/d) (n = 23,238, p-trend < 0.001). Diversity scores were based on the number of different major food groups or the number of minor food groups (subtypes) consumed within a major group. Data is presented in Table 5. (DOCX) [file pmed.1002085.s002.docx]
