# Supplementary material for: Dietary Diversity, Diet Cost, and Incidence of Type 2 Diabetes in the United Kingdom: A Prospective Cohort Study
Source: PLoS Med. 2016 Jul 19;13(7):e1002085. doi: 10.1371/journal.pmed.1002085 (PMC4951147; doi:10.1371/journal.pmed.1002085)
Supplement: S1 Table — 1 Based on reference [8] and Annex 2 on food group assignment in [22]. 2 Mixed dishes were separated into component ingredients using standardized recipes (McCance and Widdowson’s The Composition of Foods, 6th edition) [25] and assigned to food groups when ingredients in that group comprised at least 10% of the dish. (DOCX) [file pmed.1002085.s003.docx]

| **Major Food Group** | **Food group subtypes** | **Representative food items and mixed dishes^2^ on the EPIC-Norfolk Food Frequency Questionnaire** |
| --- | --- | --- |
| Dairy products | Milk | Fresh cream, milk (fortified, goat’s, sheep’s,  etc), ice cream, lasagne, quiche, meat soup  (cream of chicken) |
|  | Cheese | Cottage cheese, Brie, Cheddar, Edam, quiche,  pizza |
|  | Yoghurt | Whole milk, Greek, Fromage frais, low fat, yogurt |
|  |  |  |
| Fruit | Vitamin A-rich | Melons, peaches, dried fruit |
|  | Citrus | Oranges, grapefruit, strawberries, fruit juice |
|  | Other | Apples, pears, bananas, grapes, avocado, fruit juice, dried and tinned fruit |
|  |  |  |
| Vegetable | Vitamin A-rich | Carrots, peppers (red), oxtail soup |
|  | Dark green leafy | Spinach, broccoli, watercress, beetroot |
|  | Other | Sprouts, cabbage, cauliflower, sweet corn, mushrooms, garlic, marrow, leeks, onions, peppers (green), tomatoes, coleslaw, green salad, vegetable and meat soups, lasagne, pizza |
|  | Starchy tubers | Potatoes, chips, crisps, parsnips, potato salad, vegetable soup |
|  |  |  |
| Meat & alternatives | Flesh meat – red | Beef, burger, pork, lamb, bacon, ham, corned  beef, sausages, lasagne, meat soup (oxtail),  quiche, savoury pies |
|  | Organ meat | Liver, savoury pies (steak & kidney) |
|  | Flesh meat – poultry | Chicken/turkey, meat soup (cream of chicken |
|  | Fish & seafood | Fried fish, fish fingers, white, fish, oily fish,  shellfish, roe fish |
|  | Eggs | Eggs, quiche |
|  | Legumes (pulses), nuts & seeds | Green beans, beans, beansprouts, lentils (dry  legumes), peas, nuts, peanut butter, soya  products |
|  |  |  |
| Grains | Wholegrains | Wholemeal bread and pasta, crispbread, porridge, brown rice, cereal |
|  | Non-wholegrains (refined) | White bread, crackers, brown bread, white rice and pasta, plain biscuit, buns, cereal, pizza, quiche, lasagna, meat soup (oxtail) |
|  | | |
