# Supplementary material for: Dietary Diversity, Diet Cost, and Incidence of Type 2 Diabetes in the United Kingdom: A Prospective Cohort Study
Source: PLoS Med. 2016 Jul 19;13(7):e1002085. doi: 10.1371/journal.pmed.1002085 (PMC4951147; doi:10.1371/journal.pmed.1002085)
Supplement: S2 Table — Model 1 (n = 23,912) was adjusted for age, sex, BMI, and total energy intake (kcal/d). Model 2 (n = 23,705) as Model 1 plus the following: smoking status, total alcohol intake, physical activity level, and family history of diabetes. Model 3 (n = 23,238) as Model 2 plus the following: education and occupational social class. * p < 0.05; ** p < 0.01; *** p < 0.001. 1 All food subtypes within each food group were summed together to create a composite continuous score ranging from zero to 18 subtypes across five major food groups. (DOCX) [file pmed.1002085.s004.docx]

| **Score** | **Quintiles of the number. of food groups** | **Cases/total** | **No. of events**  **(rate per 100,000 person years)** | **Model 1** | | **Model 2** | | **Model 3** | |
| --- | --- | --- | --- | --- | --- | --- | --- | --- | --- |
|  |  |  |  | **HR** | ***95% CI*** | **HR** | ***95% CI*** | **HR** | ***95% CI*** |
|  |  |  |  |  |  |  |  |  |  |
| Diversity of all food group subtypes (0-18)^1^ | Q1 | 219/4871 | 453 | 1 |  | 1 |  | 1 |  |
|  | Q2 | 224/6410 | 352 | 0.79***** | *0.65 to 0.95* | 0.79***** | *0.65 to 0.95* | 0.79***** | *0.65 to 0.95* |
|  | Q3 | 131/3797 | 349 | 0.77***** | *0.62 to 0.97* | 0.78***** | *0.62 to 0.98* | 0.80***** | *0.64 to 0.99* |
|  | Q4 | 188/6135 | 311 | 0.68******* | *0.56 to 0.84* | 0.69****** | *0.56 to 0.85* | 0.69****** | *0.56 to 0.86* |
|  | Q5 | 88/2757 | 324 | 0.66****** | *0.51 to 0.87* | 0.67****** | *0.51 to 0.89* | 0.70***** | *0.53 to 0.92* |
|  |  |  | *P*-trend | *0.0014* |  | *0.0025* |  | *0.0057* |  |
