# Supplementary material for: Dietary Diversity, Diet Cost, and Incidence of Type 2 Diabetes in the United Kingdom: A Prospective Cohort Study
Source: PLoS Med. 2016 Jul 19;13(7):e1002085. doi: 10.1371/journal.pmed.1002085 (PMC4951147; doi:10.1371/journal.pmed.1002085)
Supplement: S3 Table — Model A adjusted for all covariables and total quantity of the dependent variable examined (n = 23,105). Model B adjusted for all covariables and waist circumference (n = 23,222). Model C adjusted for all covariables after excluding participants with HbA1c ≥ 6.5% (n = 9,914). Model D was adjusted for all covariables after excluding participants with self-reported health conditions (n = 21,110). * p < 0.05; ** p < 0.01; *** p < 0.001. (DOCX) [file pmed.1002085.s005.docx]

| **Score** | **No. of food groups** | **Model A** | | **Model B** | | **Model C** | | **Model D** | |
| --- | --- | --- | --- | --- | --- | --- | --- | --- | --- |
|  |  | **HR** | ***95% CI*** | **HR** | ***95% CI*** | **HR** | ***95% CI*** | **HR** | ***95% CI*** |
|  |  |  |  |  |  |  |  |  |  |
| Total diet dietary diversity | 0-3 | 1 |  | 1 |  | 1 |  | 1 |  |
|  | 4 | 0.86 | *0.62 to 1.19* | 0.88 | *0.63 to 1.22* | 0.64 | *0.32 to 1.24* | 0.82 | *0.58 to1.16* |
|  | 5 | 0.73 | *0.53 to 1.01* | 0.72***** | *0.52 to 0.98* | 0.53 | *0.28 to 1.00* | 0.67***** | *0.48 to 0.94* |
|  |  |  |  |  |  |  |  |  |  |
| Dairy diversity | 0 | 1 |  | 1 |  | 1 |  | 1 |  |
|  | 1 | 0.75****** | *0.61 to 0.93* | 0.72****** | *0.59 to 0.89* | 0.69 | *0.45 to 1.06* | 0.72****** | *0.58 to 0.90* |
|  | 2 | 0.81 | *0.64 to 1.03* | 0.76****** | *0.61 to 0.93* | 0.57***** | *0.37 to 0.90* | 0.75***** | *0.60 to 0.94* |
|  | 3 | 0.70***** | *0.49 to 0.99* | 0.62****** | *0.46 to 0.83* | 0.66 | *0.37 to 1.19* | 0.61****** | *0.45 to 0.84* |
|  |  |  |  |  |  |  |  |  |  |
| Fruit diversity | 0 | 1 |  | 1 |  | 1 |  | 1 |  |
|  | 1 | 0.88 | *0.67 to 1.15* | 0.92 | *0.70 to 1.20* | 0.84 | *0.48 to 1.47* | 0.95 | *0.71 to 1.27* |
|  | 2 | 0.81 | *0.62 to 1.05* | 0.84 | *0.65 to 1.07* | 0.66 | *0.40 to 1.10* | 0.81 | *0.62 to 1.06* |
|  | 3 | 0.68***** | *0.50 to 0.93* | 0.72***** | *0.55 to 0.94* | 0.42****** | *0.23 to 0.75* | 0.70***** | *0.52 to 0.95* |
|  |  |  |  |  |  |  |  |  |  |
| Vegetable diversity | 0-1 | 1 |  | 1 |  | 1 |  | 1 |  |
|  | 2 | 0.77 | *0.59 to 1.02* | 0.79 | *0.60 to 1.04* | 0.75 | *0.42 to 1.35* | 0.80 | *0.60 to 1.08* |
|  | 3 | 0.66****** | *0.51 to 0.87* | 0.72***** | *0.55 to 0.93* | 0.66 | *0.38 to 1.14* | 0.72 | *0.54 to 0.95* |
|  | 4 | 0.62****** | *0.47 to 0.84* | 0.70****** | *0.54 to 0.90* | 0.56***** | *0.32 to 0.97* | 0.67 | *0.51 to 0.89* |
|  |  |  |  |  |  |  |  |  |  |
| Meat diversity | 0 | 1 |  | 1 |  | 1 |  | 1 |  |
|  | 1 | 1.03 | *0.77 to 1.38* | 1.06 | *0.79 to 1.41* | 1.17 | *0.60 to 2.30* | 0.99 | *0.73 to 1.35* |
|  | 2 | 0.99 | *0.74 to 1.34* | 1.03 | *0.77 to 1.36* | 1.34 | *0.70 to 2.56* | 0.99 | *0.74 to 1.34* |
|  | 3 | 0.86 | *0.62 to 1.19* | 0.91 | *0.68 to 1.22* | 1.00 | *0.51 to 1.98* | 0.89 | *0.65 to 1.22* |
|  | 4 | 1.09 | *0.75 to 1.59* | 1.13 | *0.82 to 1.56* | 1.44 | *0.70 to 2.99* | 1.17 | *0.84 to 1.65* |
|  | 5-6 | 1.05 | *0.62 to 1.80* | 1.15 | *0.73 to 1.81* | 1.29 | *0.46 to 3.62* | 1.06 | *0.65 to 1.74* |
|  |  |  |  |  |  |  |  |  |  |
| Grain diversity | 0-1 | 1 |  | 1 |  | 1 |  | 1 |  |
|  | 2 | 0.99 | *0.83 to 1.20* | 0.98 | *0.82 to 1.17* | 0.96 | *0.64 to 1.43* | 0.98 | *0.81 to 1.19* |
