# Supplementary material for: Dietary Diversity, Diet Cost, and Incidence of Type 2 Diabetes in the United Kingdom: A Prospective Cohort Study
Source: PLoS Med. 2016 Jul 19;13(7):e1002085. doi: 10.1371/journal.pmed.1002085 (PMC4951147; doi:10.1371/journal.pmed.1002085)
Supplement: S4 Table — Model A adjusted for all covariables using a revised score for vegetable diversity and total diet diversity, without counting any potato items reported by EPIC participants. Model B adjusted for all covariables using a revised score for vegetable diversity and total diet diversity, counting only baked or boiled potato items reported by EPIC participants. n = 23,238. * p < 0.05; ** p < 0.01; *** p < 0.001. 1 The revised score for diversity of vegetable subtypes used the score of zero as the reference group since there were sufficient numbers at that level (n = 1,194, 5%). 2 The revised score for diversity of vegetable subtypes used the combined scores of zero and one as the reference group due to low numbers at the zero level (n = 225, 0.94%) (DOCX) [file pmed.1002085.s006.docx]

| **Score** | **No. of food groups** | **Model A** | | **Model B** | |
| --- | --- | --- | --- | --- | --- |
|  |  | **HR** | ***95% CI*** | **HR** | ***95% CI*** |
|  |  |  |  |  |  |
| Total diet dietary diversity | 0-3 | 1 |  | 1 |  |
|  | 4 | 0.87 | *0.66 to 1.15* | 0.85 | *0.62 to 1.17* |
|  | 5 | 0.67** | *0.52 to 0.88* | 0.69***** | *0.51 to 0.94* |
|  |  |  |  |  |  |
| Vegetable diversity | 0 | 1^1^ |  |  |  |
|  | 1 | 0.82 | *0.61 to 1.10* | 1^2^ |  |
|  | 2 | 0.73***** | *0.55 to 0.97* | 0.78 | *0.60 to 1.02* |
|  | 3 | 0.64** | *0.48 to 0.85* | 0.70****** | *0.55 to 0.90* |
|  | 4 | 0.72* | *0.53 to 0.97* | 0.67****** | *0.52 to 0.86* |
|  |  |  |  |  |  |
| Diversity of all food group subtypes (0-18) | Q1 | 1 |  | 1 |  |
|  | Q2 | 0.90 | *0.72 to 1.11* | 0.80* | *0.66 to 0.97* |
|  | Q3 | 0.81* | *0.68 to 0.97* | 0.78* | *0.63 to 0.98* |
|  | Q4 | 0.69** | *0.53 to 0.90* | 0.69** | *0.56 to 0.85* |
|  | Q5 | 0.77* | *0.61 to 0.97* | 0.70* | *0.53 to 0.93* |
