# Supplementary material for: Dietary Diversity, Diet Cost, and Incidence of Type 2 Diabetes in the United Kingdom: A Prospective Cohort Study
Source: PLoS Med. 2016 Jul 19;13(7):e1002085. doi: 10.1371/journal.pmed.1002085 (PMC4951147; doi:10.1371/journal.pmed.1002085)
Supplement: S5 Table — Means obtained by multivariable linear regression analysis adjusted for sex, age, and total energy intake (kcal/d) (n = 23,238). 1 All food subtypes within each food group were summed together to create a composite continuous score ranging from zero to 18 subtypes across five major food groups. (DOCX) [file pmed.1002085.s007.docx]

| **Score** | **Quintiles of the total number of food subtypes** | **Mean (£/d)** | ***95% CI*** |
| --- | --- | --- | --- |
|  |  |  |  |
| Diversity of all food group subtypes (0-18)^1^ | Q1 | 3.45 | *3.43 to 3.48* |
|  | Q2 | 3.84 | *3.82 to 3.86* |
|  | Q3 | 4.08 | *4.05 to 4.10* |
|  | Q4 | 4.39 | *4.37 to 4.41* |
|  | Q5 | 4.88 | *4.84 to 4.91* |
|  | *P-*trend | *<0.001* |  |
