# Supplementary material for: Dietary Diversity, Diet Cost, and Incidence of Type 2 Diabetes in the United Kingdom: A Prospective Cohort Study
Source: PLoS Med. 2016 Jul 19;13(7):e1002085. doi: 10.1371/journal.pmed.1002085 (PMC4951147; doi:10.1371/journal.pmed.1002085)
Supplement: S1 Protocol — (DOCX) [file pmed.1002085.s008.docx]

**Prospective Study Protocol (January 21^st^, 2015)**

**Aim (1)**: To extend the concept of food variety to include food groups beyond fruit and vegetable categories so as to examine overall diversity in a diet in relation to the aetiology of diabetes in EPIC-Norfolk; and, to improve the measurement of dietary diversity by considering variety of food subtypes (i.e. sub-major/ minor groups) within each group.

**Hypothesis**: Total food variety will be strongly associated with risk of T2D. Variety of subgroups within different main groups will also be associated with risk of T2D. Greater variety across major food groups will reduce diabetes risk, as will greater variety of subgroups within each main group.

**Methods**: Derive score from reported frequencies: a given food group is allocated a score of 0 or 1, depending on whether a food item is consumed more than once per week.

|  | Approach to assigning FFQ food items |
| --- | --- |
| Between-group diversity | Total food variety across 5  groups (0-5)  *Meat; milk; grains; fruits; and vegetables* |
| Within-group (subtype) diversity | - Meat diversity (0-6) - Dairy diversity (0-3) - Grain diversity (0-2) - Fruit diversity (0-3) - Vegetable diversity (0-4) |
| Subtypes | *Flesh meat (red); flesh meat (poultry); organ meat; fish &seafood; eggs; legumes; milk; cheese; yoghurt; whole grains; refined grains; vitamin A-rich vegetables; dark green leafy vegetables, other*  *vegetables; starchy vegetables (tubers); vitamin A-rich fruits; citrus & berry fruits; other fruits* |

Data Analysis:

1. Data cleaning and checking (e.g. remove 855 participants with prevalent diabetes)
2. Distribution of participants across diversity levels (assess low numbers for potential recoding)
3. Examine relationship between variety scores, and between each score and total energy intake
   1. Pearson correlation matrices of scores for variety
   2. Mean energy intake by the number of food groups consumed in each variety score
4. Examine the social patterning of derived scores for total food variety across five and seven food groups.
   1. Crude bivariate associations: mean scores across categories of gender, age, education, social class, marital status and lifestyle factors
   2. Regressions of socio-demographic constructs against diversity scores, adjusted for energy, age, sex and smoking status.
5. Aetiological association of total food and subgroup variety with type 2 diabetes
   1. Multivariable Cox proportional hazards models to estimate the rate ratios and 95% CIs of developing T2D (primary outcome: new cases) for each additional food group and subgroup, compared with the lowest score (reference category). Co-variables include: age, sex, family history, BMI, PA, Kcal, smoking status, SES (education, social class)
   2. Main analyses: (i) A single diversity score (+ co-variables); and, (ii) Mutual adjustment of all diversity scores (+ co-variables)
   3. SA: (i) add quantity; (ii) add waist circumference; (iii) exclude participants with chronic illnesses; (iv) exclude participants with undiagnosed diabetes (Hb1Ac ≥6.5); (v) alternative scoring for vegetable items (exclude any potato; count only boiled or baked potato)

**Aim (2)**: To estimate the total diet cost associated with greater dietary diversity between and within food groups.

**Hypothesis**: Higher diet diversity will be associated with higher total diet cost.

**Methods**:

Data Analysis:

1. Univariate analysis of total diet cost
2. Bivariate analysis of diet diversity scores and diet cost
3. Bivariate associations of diet cost across gender, age, education, social class, marital status and lifestyle factors
4. Cross-sectional association of total diet and within-group diversity with total diet cost, at baseline
   1. Multivariable linear regression, adjusting for age, sex, and total energy intake.
   2. Post-estimation of regression coefficients to calculate adjusted means (95% CI)
